# Glycosyltransferase efficiently controls phenylpropanoid pathway

**DOI:** 10.1186/1472-6750-8-25

**Published:** 2008-03-05

**Authors:** Anna Aksamit-Stachurska, Alina Korobczak-Sosna, Anna Kulma, Jan Szopa

**Affiliations:** 1 Faculty of Biotechnology, Wroclaw University, Przybyszewskiego 63/77, 51-148 Wroclaw, Poland; 2 Faculty of Biological Sciences, Wroclaw University, Przybyszewskiego 63/77, 51-148 Wroclaw, Poland

## Abstract

**Background:**

In a previous study, anthocyanin levels in potato plants were increased by manipulating genes connected with the flavonoid biosynthesis pathway. However, starch content and tuber yield were dramatically reduced in the transgenic plants, which over-expressed dihydroflavonol reductase (DFR).

**Results:**

Transgenic plants over-expressing dihydroflavonol reductase (DFR) were subsequently transformed with the cDNA coding for the glycosyltransferase (UGT) of Solanum sogarandinum in order to obtain plants with a high anthocyanin content without reducing tuber yield and quality. Based on enzyme studies, the recombinant UGT is a 7-O-glycosyltransferase whose natural substrates include both anthocyanidins and flavonols such as kaempferol and quercetin. In the super-transformed plants, tuber production was much higher than in the original transgenic plants bearing only the transgene coding for DFR, and was almost the same as in the control plants. The anthocyanin level was lower than in the initial plants, but still higher than in the control plants. Unexpectedly, the super-transformed plants also produced large amounts of kaempferol, chlorogenic acid, isochlorogenic acid, sinapic acid and proanthocyanins.

**Conclusion:**

In plants over-expressing both the transgene for DFR and the transgene for UGT, the synthesis of phenolic acids was diverted away from the anthocyanin branch. This represents a novel approach to manipulating phenolic acids synthesis in plants.

## Background

**Figure 1:**
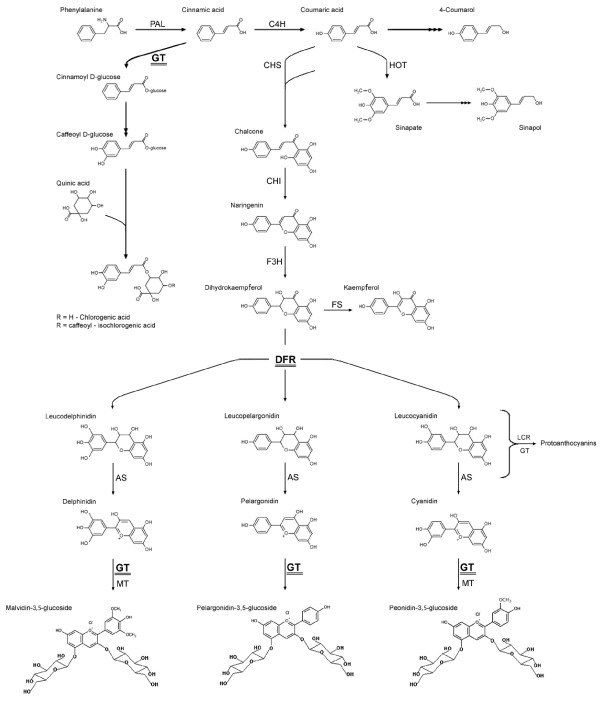
**Scheme of the phenylpropanoid pathway**. L-phenylalanine ammonia lyase (PAL), cinnamate 4-hydroxylase (C4H), chalcone synthase (CHS), chalcone isomerase (CHI), dihydroflavonol 4-reductase (DFR), flavanone 3-hydroxylase (F3H), flavonol synthase (FS), glucosyltransferase (GT), hydroxylase-O-methyl transferase (HOT), methyltransferase (MT), anthocyanin synthase (AS), and leucoanthocyanidin reductase (LCR).

The phenylpropanoid pathway is the source of numerous phenylalanine derivatives. The main branches of the phenylpropanoid pathway are presented in Figure 1. The main products of the various branches are lignin, flavonoids, chlorogenic acid, salicylic acid and catecholamines. Of the compounds synthesized by the phenylpropanoid pathway, the most abundant is lignin, which makes up 66.0% of the total, followed by proanthocyanins (18.6%), phenolic acids (13.0%), anthocyanins (2.5%), salicylic acids (0.4%), and catecholamines (0.4%). Most of the compounds synthesized by the phenylpropanoid pathway can be glycosylated by glycosyltransferases. Glycosylation increases solubility, reduces reactivity, and increases stability.

The components of this pathway play an important role in plant physiology. Flavonoids represent a large class of phenylpropanoids. Flavonoids serve as pigments, which attract pollinators and protect the plant from UV irradiation. Flavonoids also act as antioxidants, photoreceptors, antimicrobials, feeding deterrents, and metal chelators [1].

The phenylpropanoid pathway begins with the deamination of phenylalanine to cinnamic acid. In subsequent reactions, hydroxycinnamoyl CoA thioester is formed. This compound is the substrate for branch pathways responsible for the synthesis of lignin monomers, anthocyanins, coumarins and chlorogenic acid [2].

Among the products of the phenylpropanoid pathway are flavanones, which are converted to kaempferol or anthocyanidins in several steps involving hydroxylation. These products are then glycosylated by a specific glycosyltransferase. In potatoes, the two main anthocyanins are pelargonidin and peonidin, which have a tri-saccharide side chain attached to the 3-hydroxy group of the aglycone [3].

In a previous study, anthocyanin levels in potato plants were modified by manipulating genes connected with the flavonoid biosynthesis pathway [3]. The genes manipulated were those coding for the key enzymes in the pathway: chalcone synthase (CHS), chalcone isomerase (CHI), and dihydroflavonol reductase (DFR). The plants were transformed using constructs containing one, two or all three of these genes. The constructs contained the cDNA sequences inserted in either the sense or anti-sense orientation. The selected transgenic plants were then analyzed for anthocyanin content and antioxidant capacity. The construct that was most effective in increasing anthocyanin synthesis was the single-gene construct containing the cDNA sequence for DFR in the sense orientation. The construct which was most effective in repressing anthocyanin biosynthesis was the single-gene construct containing the cDNA sequence for DFR in the anti-sense orientation. In the plants carrying the transgene for DFR, anthocyanin content was perfectly correlated with antioxidant capacity. However, in all of the transgenic plants, starch content was lower than in the control. Depending on the construct used, starch content was reduced from a few percent to over 90%. The reduction in starch content was accompanied by a significant reduction in tuber yield. This suggests that high concentrations of flavonoids suppress carbohydrate synthesis, thereby reducing tuber production.

Glycosylation reduces the reactivity and increases the stability of flavonoids. Recently, transgenic potato plants which over-produce glycosyltransferase (UGT) have been produced and characterized [4]. In tuber extracts from plants which over-express the gene for UGT, the level of 3,5-O-substituted anthocyanidins was significantly higher than in tuber extracts from the control plants, but significantly lower than in tuber extracts from plants bearing the transgene for DFR. Of the six anthocyanins identified, the ones present in the highest amounts were pelargonidin 3-rutinoside-5-glucoside acylated with p-coumaric acid and peonidin 3-rutinoside-5-glucoside acylated with p-coumaric acid. Glycosylated flavonoids do not reduce starch content and tuber yield.

In order to substantially increase flavonoid content without reducing tuber yield and quality, plants over-expressing the transgene for DFR were super-transformed with cDNA coding for the UGT of *Solanum sogarandinum *under the control of the tuber-specific B33 promoter.

It was expected that the level of anthocyanin synthesis would be at least the same as in the initial plants, and that starch content and tuber yield would not be reduced because of the higher levels of anthocyanins. In the super-transformed plants, tuber production was much higher than in the initial plants bearing only the transgene coding for DFR, and was almost the same as in the control plants. The anthocyanin level was lower than in the initial plants, but still higher than in the control plants. Unexpectedly, the super-transformed plants also produced large amounts of kaempferol, chlorogenic acid, isochlorogenic acid, sinapic acid and proanthocyanins. In plants over-expressing both the transgene for DFR and the transgene for UGT, the synthesis of phenolic acids was diverted away from the anthocyanin branch. This represents a novel approach to manipulating phenolic acids synthesis in plants. Chlorogenic acid is a strong antioxidant which reduces the oxidation of low-density lipids and may prevent carcinogenesis. Increasing dietary intake of chlorogenic acid should therefore be beneficial to consumer health.

## Results

### The substrate specificity

**Figure 2:**
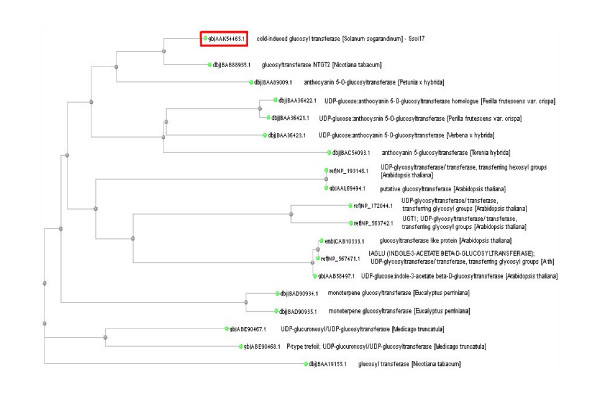
**Mlecular phylogenetic tree based on deduced amino acid sequences of plant glycosyltransferases**. Amino acid sequences of plant glycosyltransferases were obtained by using Blast program with Blosum62 algorithm. The tree was constructed by the Fast Minimum Evolution method. The dendrogram was created using the Clustal Sequence Alignment software package.

The glycosyltransferase gene used in this study was isolated from several cold-induced clones of the cold-resistant potato species *Solanum sogarandinum *[5]. The product of this gene was 83% similar to the flavonoid 7-O-glycosyltransferase of *Nicotiana tabacum*, 68% similar to the anthocyanin-5-O-glycosyltransferase of *Petunia hybrida*, 60% similar to the UGT of *Perilla frutescens*, 57% similar to the UGT of *Verbena hybrida*, and 53% similar to the UGT of *Torenia hybrida*. A molecular phylogenetic tree based on the deduced amino acid sequence of the UGTs of several plant species is presented in Figure 2.

In order to study the expression of UGT, the cDNA encoding for it was inserted into E. coli using the pQE 30 vector. The recombinant protein was purified by affinity chromatography and subjected to enzymatic analysis. As stated above, the UGT of *S. sogarandinum *is highly similar to the flavonoid 7-O-glycosyltransferase of *N. tabacum *and the anthocyanin-5-O-glycosyltransferase of *P. hybrida*. Therefore, the recombinant protein was tested using several compounds that serve as substrates for both the tobacco and petunia enzymes.

**Figure 3:**
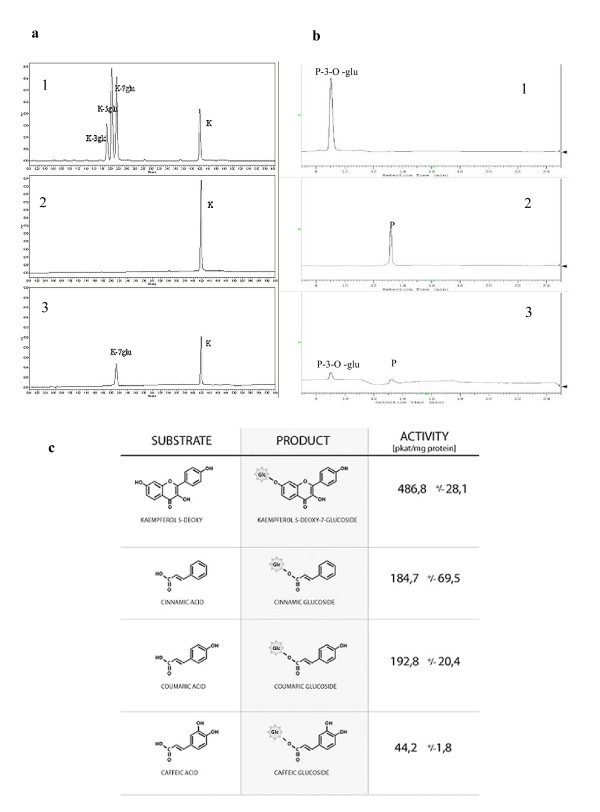
**Analysis of specificity of recombinant UGT**. a) Analysis of specificity of recombinant UGT by UPLC, 1.: Standards of kaempferol glucosides and kampferol. 2.:Standard of kaempferol 3.: Chromatogram of standard kaempferol incubated with recombinant glycosyltransferase. Incubation with recombinant UGT, UDP-glucose, and keampferol produced a major product with the expected retention time and UV spectrum of keampferol-7-O-glucoside. The enzyme assay and product analysis is described in the Methods section. b) Analysis of specificity of recombinant UGT by HPLC. 1.: Standard of peonidin-3-O-glucoside, 2.: Standard of peonidin chloride., 3.: Chromatogram of standard peonidin chloride incubated with recombinant glycosyltransferase. Incubation with recombinant UGT, UDP-glucose, and peonidin chloride produced a major product with the expected retention time and UV spectrum of peonidin-3-O-glucoside. The enzyme assay and product analysis is described in the Methods section. c). Substrate specificity of recombinant UGT. The substrate specificity was measured as described in the Methods section with the use of UDP-Glu as a glucose donor. The mean value (n = 4) ± SE is presented.

Of the substrates tested, (cinnamic acid, caffeic acid, coumaric acid, kaempferol, kaempferol-3-O-glycoside, kaempferol-5-O-glycoside, kaempferol-7-O-glycoside, 5-deoxykaempferol, peonidin chloride and peonidin-3-O-glycoside) kaempferol was the substrate most effectively glycosylated by the recombinant protein (Fig. 3a, 3b). The products of the reaction were separated and identified using HPLC or UPLC. In a previous study [4], this enzyme was predicted to be anthocyanin 5-O-glycosyltransferase. However, further enzyme studies revealed it to be a flavonoid 7-O-glycosyltransferase that also had anthocyanin 3- and 5-O-glycosyltransferase activity. The activity of the recombinant protein on various phenolic compounds is presented in Figure 3c. With cinnamic acid and coumaric acid, the reaction rate was one-fourth the rate with kaempferol. With caffeic acid and chlorogenic acid, only trace activity was observed. Kinetic parameters were calculated based on the Lineweaver-Burk plot using kaempferol as the substrate (Table 1). The Km was 8.5 μM for kaempferol, and 21.4 μM for peonidin. This indicates that the recombinant protein is a 7-O-glycosyltransferase. In another study, the 5-O-glycosyltransferase from *Dorotheanthus bellidiformis *was highly homologous to the glycosyltransferases of several solanaceous plants and could also glycosylate flavonols such as quercetin [6]. This suggests that the natural substrates of the UGT of *S. sogarandinum *include both anthocyanidins and flavonols such as kaempferol and quercetin.

**Table 1:** Substrate specificity of recombinant UGT.

**Substrates**	**Km (μM)**	**Vmax (μmol/s × mg)**	**Vmax/Km**
**peonidin**	21.42 ± 1.52	478.00 ± 138.12	22.35
**Kaempferol**	8.5 ± 0.62	268.685 ± 36.31	31.36

The substrate specificity was measured as described in the Methods section with the use of UDP-Glu as a glucose donor. The mean value (n = 4) ± SE is presented.

### Generation and selection of transgenic plants

**Figure 4:**
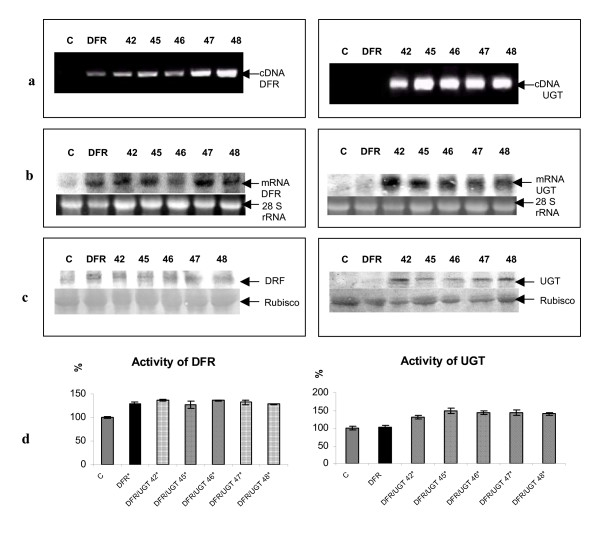
**Selection of transgenic plants**. a) Agarose gel electrophoresis of PCR products obtained with primers for DFR (1143 bp) cDNA and UGT (1100 bp) cDNA on genomic DNA isolated from tissue-cultured potato plants. C: negative control (non-transformed plant). DFR: transgenic plant over-expressing DFR. Transgenic lines over-expressing DFR and UGT are numbered. b) Northern blot analysis of RNA isolated from tubers of control plant (marked C), tubers of transgenic plant over-expressing DFR and tubers of independent transgenic lines over-expressing DFR and UGT (numbered). 50 μg of total RNA was loaded onto each lane, and the first blot was hybridized with ^32^P- labeled DFR cDNA and the second blot was hybridized with ^32^P- labeled UGT cDNA Bottom panel shows ribosomal RNA stained with ethidium bromide as a control of RNA applied onto the gel. c) Western blot analysis of protein extract isolated from the potato tuber. Blots were probed with anti DFR antibodies (left) and anti UGT antibodies (right). C: negative control (non-transformed plant). DFR: transgenic plant over-expressing DFR. Transgenic lines over-expressing DFR and UGT are numbered. Bottom panel is the ribulose-1,5 bisphosphate carboxylase/oxygenase (Rubisco) on the same blot stained with Pounceau red. d) Activity of UGT and DFR in transgenic and wild type potato plants DFR and UGT over-expressing plants (numbered-striped bars) were analyzed and compared with transgenic plant over-expressing DFR (DFR- black bar) and with the control (C- grey bar). *Asterisks *(*) indicate values that are significantly different from the wild type plants with p > 0.05.

In order to produce transgenic potato plants with increased levels of stable glycosylated anthocyanins, two-week-old leaf explants of transgenic potato line DFR 11 [3] were subsequently super-transformed by dipping them in a suspension of Agrobacterium tumefaciens carrying the cDNA sequence coding for the UGT of *S. sogarandinum *(GenBank accession number AY033489). This transgene was placed under the control of the tuber-specific B33 promoter. The regenerated plants were then screened using PCR with primers specific for the gene for dihydroflavonol 4-reductase and for glycosyltransferase (Fig. 4a). Those with the highest signal were selected for further analysis. Further selection was carried out using northern and western blotting. Expression of both transgenes was high in all of the lines selected (Fig. 4b,c). The selected super-transformed lines were subjected to further selection based on the activity of both DFR and UGT (Fig 4d). DFR activity in the super-transformed plants was as high as it was in line DFR 11. This indicates that the second round of transformation had no effect on DFR activity. UGT activity in the super-transformed plants was measured using kaempferol as the substrate. In lines DFR/UGT 42, 45, 46, 47 and 48, UGT activity was from 20% to 50% higher than in the control. These lines were therefore selected for further analysis.

### Phenotypical analysis

**Figure 5:**
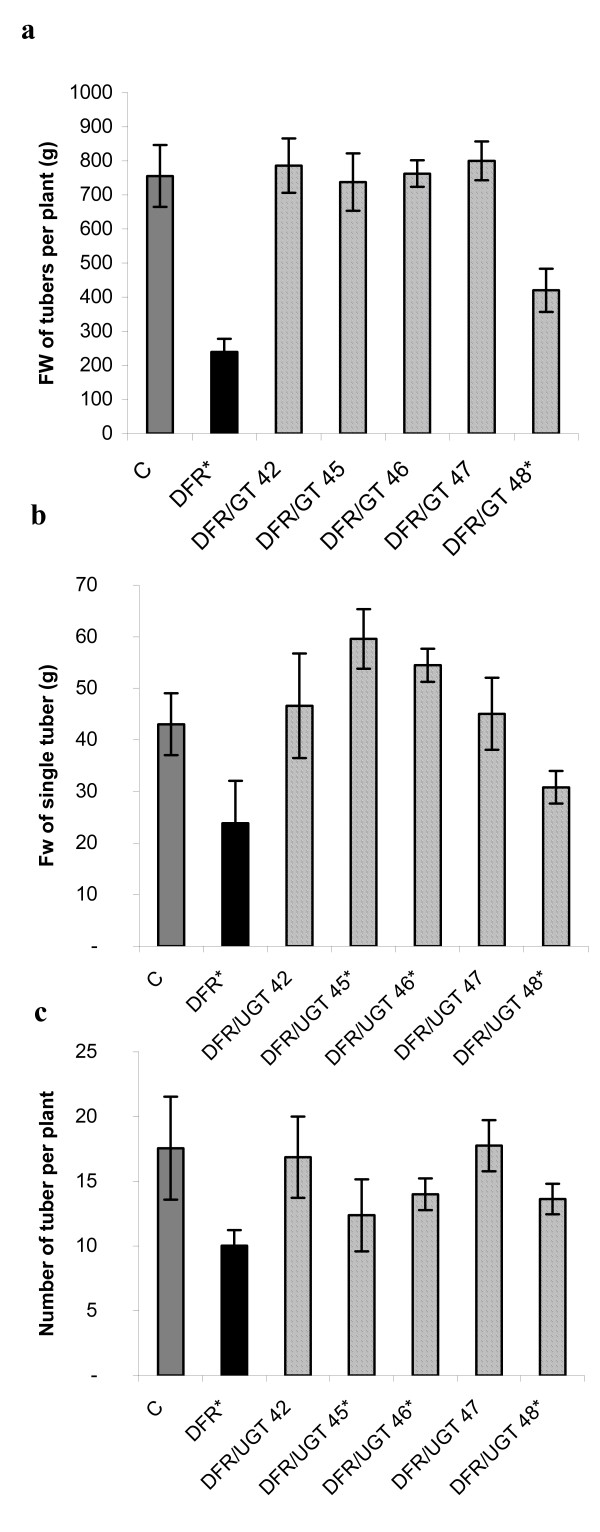
**The yield of field cultivated transgenic and control plants**. Tubers from 10 plants were collected and used for analysis. Tubers from independent transgenic lines over-expressing DFR and UGT (numbered) were analyzed and compared to the control (C) and to transgenic line over-expressing DFR (DFR) from field trials performed in 2005. Grey bar- control, black bar- transgenic plant over-expressing DFR, striped bars- transgenic lines over-expressing DFR and UGT. *Asterisks *(*) indicate values that are significantly different from the wild type plants at p > 0.05.

In the super-transformed plants, the above-ground parts of the plant were generally the same in size and shape as in line DFR 11 and the control. The only exception was flower color, which was white in the super-transformed plants. This suggests either that they contained less anthocyanins, or that they accumulated colorless compounds such as leucoanthocyanins. In line DFR 11, tuber production is lower than in the control [3]. In all the super-transformed plants, tuber production was increased compared to DFR 11 and was about the same as in the control except in line 48, in which it still remained significantly lower (Fig. 5a). The tuber size was also about the same as in the control in lines 42 and 47 while in lines 45 and 46 it was larger. In contrast, like DFR 11, the line 48 produced smaller tubers than the control (Fig. 5b). The number of tubers per plant was the same as the control in lines 42 and 47, and lower than the control in lines 45, 46, and 48. Nevertheless, this was still higher than in the single DFR transformant (Fig. 5c). This suggests that the over-expression of the gene coding for UGT cancels out the reduction in yield caused by the over-expression of the gene coding for DFR.

### Anthocyanins content in tuber extracts

**Figure 6:**
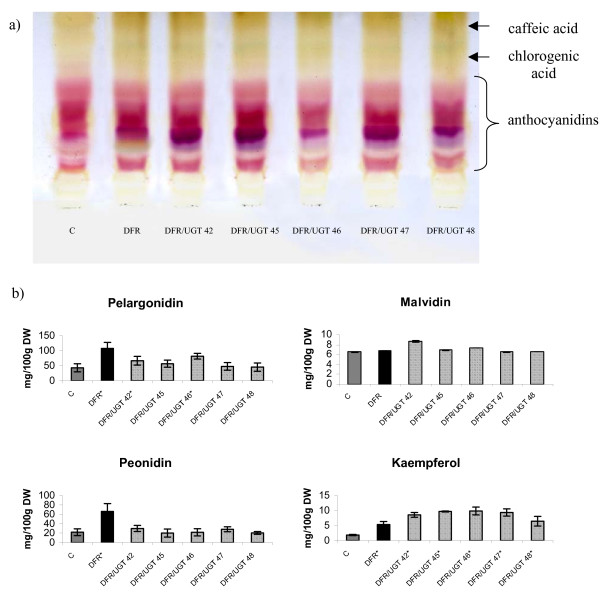
**Determination of anthocyanins content in epidermal tuber extracts**. a) TLC chromatogram of separated fractions of anthocyanidins from epidermal tuber extracts of control (C), transgenic plant over-expressing DFR (DFR) and transgenic lines over-expressing DFR and UGT (numbered). As a mobile phase 1-butanol-acetic acid-water (4:1:5) was used. b) HPLC determination of anthocyanins content in epidermal tuber extracts from control (C-grey bar), transgenic plant over-expressing DFR (DFR-black bar) and transgenic lines over-expressing DFR and UGT (numbered-striped bars). The mean value (n = 6) ± SE is presented. *Asterisks *(*) indicate values that are significantly different from the wild type plants at p > 0.05.

The levels of flavonoids in the tubers of the super-transformed plants was also measured using thin layer chromatography and were found to be higher than in the control plants (Fig. 6a). Anthocyanins content was also measured using HPLC in order to determine the effect of the two transgenes. As expected, elevated levels of anthocyanins were detected in the super-transformed plants (Fig. 6b). The anthocyanins found in the highest amounts were the trisaccharide derivatives of pelargonidin (pelargonidin 3-rut 5-glu acylated with p-coumaric acid), peonidin (peonidin 3-rut 5-glu acylated with p-coumaric acid) and malvidin (malvidin 3-rut 5-glu acylated with p-coumaric acid). This confirmed the results obtained by TLC analysis. The level of pelargonidin glycoside was significantly higher in two super-transformed lines than in the control. In line 46, the level was more than twice as high as in the control. In line 42, the level was 1.5 times higher than in the control. Nevertheless, this was a reduction compared to the pelargonidin level found in line DFR 11, in which it was more than three times higher than in the control. In the other super-transformed lines, the level of pelargonidin glycosides was also higher than in the control, although the difference was not statistically significant. The malvidin content was slightly but insignificantly higher in lines 42, 45 and 46 than in the control. The level of peonidin glycoside was higher than the control in only two of the super-transformed lines. In lines 42 and 47, the level of peonidin glycoside was about 1.3 times higher than in the control but the changes were not statistically significant. Considering that UGT is highly homologous to flavonoid 7-O-glycosyltransferase, the levels of anthocyanin precursors were also measured. Kaempferol content was more than four times higher in lines 42, 45, 46, and 47 and almost four times higher in line 48 than in the control. Except of line 48, this was an almost two-fold increase in the amount of kaempferol in the super-transformed lines compared to DFR 11 (Fig. 6b). This suggests that 7-O-glycosylated kaempferol is a less suitable substrate for DFR than non-glycosylated kaempferol. The increase in UGT activity therefore increased flavonol accumulation, thereby suppressing anthocyanin synthesis. This might explain why the amount of accumulated anthocyanins in the super-transformed lines was less than half as much as in line DFR 11.

### Phenolic acid, lignin and proanthocyanins content in tuber extract

**Figure 7:**
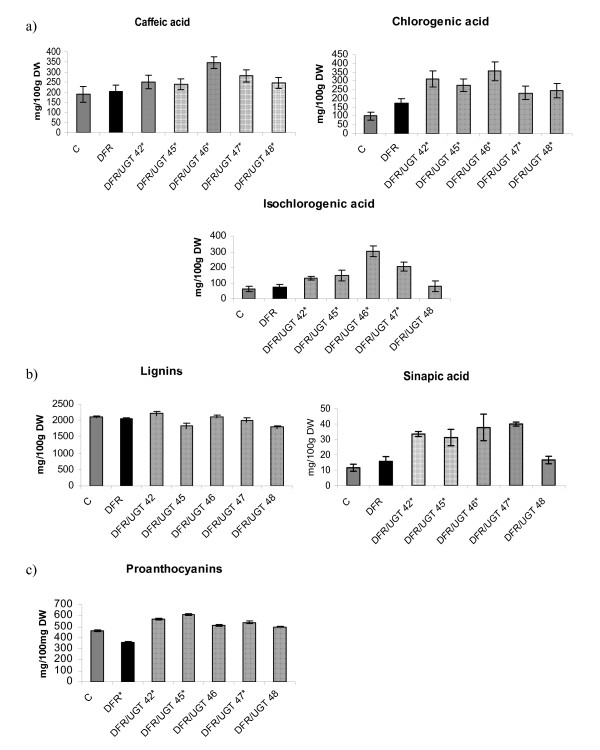
**Phenolic acid, lignin and proanthocyanins content in tuber extract**. a) HPLC determination of phenolic acid in epidermal tuber extracts from control (C-grey bar), transgenic plant over-expressing DFR (DFR-black bar) and transgenic lines over-expressing DFR and UGT (numbered-striped bars). The mean value (n = 6) ± SE is presented. *Asterisks *(*) indicate values that are significantly different at p > 0.05 from the wild type plants. b) Determination of lignin and sinapic acid content in epidermal tuber extracts from control (C-grey bar), transgenic plant over-expressing DFR (DFR-black bar) and transgenic lines over-expressing DFR and UGT (numbered-striped bars). The mean value (n = 6) ± SE is presented. *Asterisks *(*) indicate values that are significantly different (with p > 0.05) from the wild type plants. c) Determination of proanthocyanins content in epidermal tuber extracts from control (C-grey bar), transgenic plant over-expressing DFR (DFR-black bar) and transgenic lines over-expressing DFR and UGT (numbered-striped bars). The mean value (n = 6) ± SE is presented. *Asterisks *(*) indicate values that are significantly different from the wild type plants at p > 0.05.

In plants, chlorogenic acid is synthesized using three different pathways. In the glycosylation pathway, the first step is the glycosylation of the carboxyl group of cinnamic acid [7]. The product of this reaction is then converted into an activated intermediate, caffeoyl D-glucose. This intermediate is then combined with quinic acid to yield chlorogenic acid. Therefore, the levels of phenolic acids in the super-transformed lines were measured and found to be significantly higher in most lines than in the control (Fig. 7a). The level of isochlorogenic acid was as much as six times higher than in the control, as was the case with line 46. The level of chlorogenic acid was two to three times higher than in the control. The level of caffeic acid was 1.3 times higher than in the control. When assayed *in vitro*, the recombinant protein had a low affinity for cinnamic acid. Nevertheless, the affinity *in vivo *may be high enough to ensure adequate synthesis of chlorogenic acid by the glycosylation pathway.

Phenolic acids serve as substrates in lignin biosynthesis. Lignin is composed of monomer precursors called monolignols. The main step in lignin formation is the conversion of cinnamic acid into p-coumaryl alcohol, coniferyl alcohol and sinapyl alcohol. In the super-transformed lines, the level of sinapic acid glycoside was higher than in the control in all analyzed lines except line 48. Nevertheless, lignin content was not higher (Fig. 7b).

The super-transformed plants had white flowers, which suggest that colorless compounds were accumulated. The first product of DFR activity is leucoanthocyanidin, which is colorless. Leucoanthocyanidin is subsequently converted to proanthocyanin, which is also colorless. This conversion is effected by an enzyme, which is similar to DFR acting together with glycosyltransferase [8]. Therefore, the level of proanthocyanins in the super-transformed plants was measured and found to be higher in all of the super-transformed lines than in the control (Fig. 7c). The differences were statistically significant except in lines 46 and 48. In line 45, the level of proanthocyanins was 31% higher than in the control. In line 46, the level of proanthocyanins was only 7% higher than in the control. On the other hand, in line DFR 11, the level of proanthocyanins was 22% lower than in the control. This indicates that the expression of the gene for UGT changes the flux of substrates and precursors in the phenylpropanoid pathway.

### Antioxidant capacity in tuber extracts

**Figure 8:**
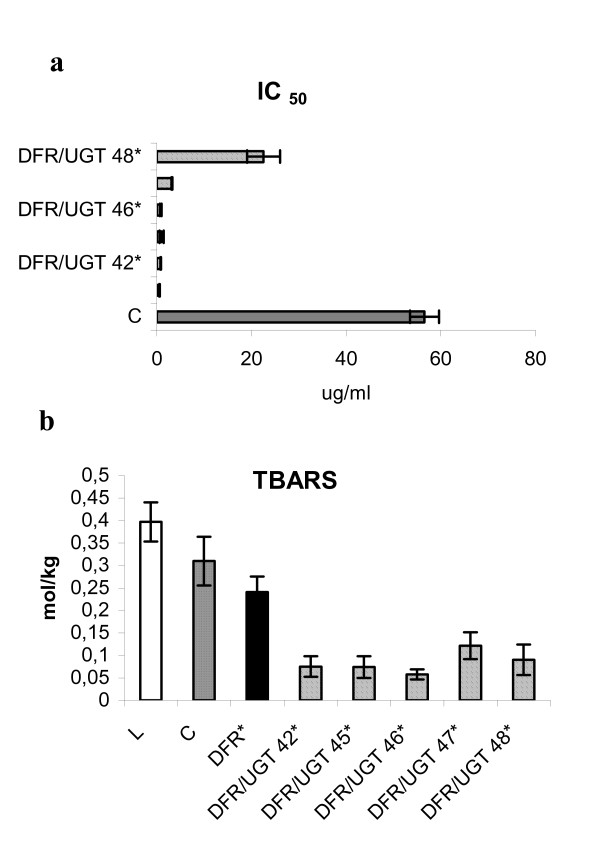
**Antioxidant potential of potato tuber extract**. a) The antioxidant potential (presented as IC_50_) of potato extract from the control (C), DFR- transgenic plant over-expressing DFR and transgenic lines over-expressing DFR and UGT (numbered). The analysis of potato extracts was performed as specified in Methods. Grey bar- control, black bar- transgenic plant over-expressing DFR, striped bars- transgenic lines over-expressing DFR and UGT. The mean values from three independent measurements + confidence interval is presented. Asterisks (*) indicate values that are significantly different at p > 0.05 from the wild type plants. b) TBARS formation in Linola flax oil in the presence of potato extract. Crude oil extracted from Linola seeds alone or supplemented with potato extract from the control (C), transgenic plant over-expressing DFR (DFR) and transgenic lines over-expressing DFR and UGT (numbered) was heated for 40 min at 140°C. Data are mean TBARS levels from six repetitions (two independent lipid extractions) measured spectrophotometrically at 535 nm. White bar- oil extracted from Linola seeds alone, grey bar- oil supplemented with extract of control, black bar- oil supplemented with extract of transgenic plant over-expressing DFR, striped bars- oil supplemented with extract of transgenic lines over-expressing DFR and UGT. *Asterisks *(*) indicate values that are significantly different (with p > 0.05) from the wild type plants.

Antioxidant compounds enable plants to cope with adverse environmental conditions, including low temperatures, UV radiation, microbial infection, and infestation by pests. Among the antioxidant compounds found in plants are phenolic acids such as chlorogenic acid, isochlorogenic acid, caffeic acid, and anthocyanins. All of these compounds help protect plants from environmental stress. They also help maintain oxidative status in potato tubers [3]. Therefore, the total antioxidant capacity was measured in tuber extracts from the super-transformed plants. Results were recorded in terms of IC50, which represents the amount of extract required to inhibit luminol luminescence by 50%. The total antioxidant capacity was from 60 to 98% higher in the super-transformed lines than in the control (Fig. 8a). The correlation coefficient for IC50 and phenolic acids content was -0.82. The correlation coefficient for IC50 and anthocyanins content was -0.72. This suggests that phenolic acids and anthocyanins affect antioxidant capacity to about the same degree.

### Antioxidative protection of polyunsaturated fatty acids by tuber extracts

Flavonoids and phenolic acids are powerful antioxidants. This suggests that they may prevent lipid oxidation both *in vivo *and *in vitro*. They may therefore improve the storability and quality of oil [9].

Therefore, tuber extracts from the super-transformed plants were tested in terms of their ability to prevent peroxidation of polyunsaturated fatty acids in flaxseed oil. Lipid peroxidation was evaluated by measuring the level of thiobarbituric acid-reactive substances (TBARS). In extracts from the super-transformed lines, the level of TBARS was from 60% to 85% percent lower (Fig. 8b). This is about twice as much as with the extract from line DFR 11. The correlation coefficient for antioxidant capacity and phenolic acids content was -0.70. The correlation coefficient for antioxidant capacity and anthocyanins content was -0.71.

## Discussion

Secondary metabolites are very important constituents of plant cells. The phenylpropanoid pathway is the source of large number of phenylalanine derivatives [1,10]. Deamination of phenylalanine by phenylalanine-ammonia lyase yields trans-cinnamic acid, which is then hydroxylated to give 4-coumarate. This is the starting point for several very important biochemical pathways in plants. In potatoes, the main products of the various branches of the phenylpropanoid pathway are lignin, flavonoids, chlorogenic acid, salicylic acid and catecholamines. Lignin, flavonoids and phenolic acids are the most abundant products. Lignin synthesis increases in response to microbial infection. Lignin is the second most abundant macromolecule in the biosphere, surpassed only by cellulose. Lignin enhances pressure resistance in plant cell walls [11]. Interest in flavonoids and phenolic acids is increasing because some of them have been found to have antioxidant, anti-allergenic, anti-viral or anti-inflammatory properties [12,13].

Flavonoids protect plants from UV irradiation [14]. They also chelate metals. Phenolic acids are the most powerful antioxidants found in plants [15]. Increasing the activity of the phenylpropanoid pathway in crop plants may prove useful in increasing resistance to biotic and abiotic stressors. It may also help in producing fruits and vegetables with higher levels of antioxidants, which would increase storability and nutritional value.

Genetic transformation is a powerful method for improving crop plants. In recent studies, we have generated transgenic plants that over-express genes coding for key enzymes involved in flavonoid synthesis. In one of these studies, anthocyanin production was found to be greatly increased in transgenic potato plants that over-express DFR [3]. However, the increase in anthocyanin content was accompanied by a significant decrease in starch content and tuber yield. High levels of anthocyanins may therefore inhibit carbohydrate synthesis. The reactivity of anthocyanins can be reduced by glycosylation. In one study on potatoes, over-expression of UGT increased the level of glycosylated anthocyanidins [4]. In the present study, transgenic potato plants bearing the cDNA sequence for DFR were super-transformed with the cDNA sequence for UGT. It was expected that this would substantially increase flavonoids content without severely reducing starch content and tuber yield. The UGT of *S. sogarandinum *is highly homologous to enzymes found in other plants, in particular the flavonoid 7-O-glycosyltransferase of *N. tabacum *and the anthocyanin-5-O-glycosyltransferase of *P. hybrida*. The chief substrate of flavonoid 7-O-glycosyltransferase is kaempferol [16]. However, the specificity of a recombinant protein cannot be reliably predicted on the basis of sequence homology alone.

In this study, the activity of the recombinant UGT was therefore tested in vitro using different substrates. The recombinant UGT proved to glycosylate a spectrum of substrates. However, it was more effective in glycosylating flavonols such as kaempferol than in glycosylating anthocyanidins. Based on enzyme kinetics, the recombinant UGT preferentially glycosylates the 7-OH group of flavonols. Unexpectedly, the level of glycosylated anthocyanidins was lower in the super-transformed lines than in line DFR 11. This may be due to competition for substrate, or it may be due to changes in the flow of compounds in the different branches of the phenylpropanoid pathway, a phenomenon which has been recently reported [17]. In the tubers of elicitor-treated potatoes, decreased synthesis of chlorogenic acid is accompanied by increased synthesis of products from other branches of the phenylpropanoid pathway, for example, hydroxycinnamoyltyramine and feruloyltyramine. In another study, on the other hand, manipulating hydroxycinnamoyl-CoA quinate hydroxycinnamoyl transferase affected the level of chlorogenic acid without affecting the levels of other soluble phenolics [7].

The level of kaempferol and its derivatives was also measured in tuber extracts of the super-transformed lines. The level of kaempferol glycoside was four times higher than in the control. The glycosylation of kaempferol therefore increased its accumulation and decreased its flux to further steps of anthocyanin biosynthesis.

Of the three pathways involved in the synthesis of phenolic acids, the glycosylation pathway is the most effective. Although the recombinant enzyme had a very low affinity for cinnamic acid in vitro, the levels of phenolic acid were very high in tuber extracts from the super-transformed lines. The level of chlorogenic acid was up to three times higher than in the control. This suggests that the affinity of the recombinant UGT for cinnamic acid in vivo is high enough to ensure adequate chlorogenic acid synthesis. The genes coding for the key enzyme of the glycosylation pathway are of particular interest because of the high bioavailability of chlorogenic acid.

Based on studies using transgenic plants, the key enzyme of the glycosylation pathway was identified as hydroxycinnamoyl-CoA quinate hydroxycinnamoyl transferase (HQT). In transgenic tomato plants, which over-express the gene for HQT, the level of chlorogenic acid is up to 85% higher than in control plants [7]. In the present study, the level of chlorogenic acid in the super-transformed plants was even higher, three times higher than in the control. The regulation of anthocyanin biosynthesis appears to be similar to the regulation of proanthocyanin biosynthesis. This conclusion is based on several lines of evidence:

• in extracts of sainfoin (*Onobrychis viciifolia*), dihydroflavonol reductase (DFR) and leucoanthocyanidin reductase (LR) are active as a multi-enzyme complex [18];

• in barley, DFR is inhibited by leucoanthocyanidin [19];

• in transgenic *Lotus corniculatus *plants carrying the Sn gene from maize, decreased proanthocyanidin levels were accompanied by decreased DFR and LR activities [20]; and

• grains carrying a mutation in the Ant 19 gene accumulate approximately 10% wild type proanthocyanin and have dramatically reduced DFR and LR activities [19].

Leucoanthocyanins, the immediate product of DFR, are therefore converted primarily to proanthocyanins when the glycosylation pathway is stimulated by the over-expression of UGT.

Compared to the control, antioxidant capacity was induced to about the same degree both in the super-transformed lines and in line DFR 11. However, lipid peroxidation was more effectively prevented by tuber extracts from the super-transformed lines than by the extract from line DFR 11.

As expected, glycosylated intermediates of the phenylpropanoid pathway were less biologically active than their non-glycosylated counterparts, and therefore did not reduce starch content (data not shown) and tuber yield. In the super-transformed lines, starch content and tuber yield were about the same as in the control.

Simultaneous over-expression of the genes for DFR and UGT increases biosynthesis of kaempferol, anthocyanins and phenolic acids. However, the increase in flavonols is mostly due to the specificity of UGT, which prefers kaempferol as a substrate. Further study is needed to elucidate why the increase in phenolic acids content is much greater in transgenic plants bearing the genes for both DFR and UGT than in transgenic plants bearing the gene for either of these enzymes alone.

## Conclusion

In transgenic plants simulanious expression od dihydroflavonol reductase and glycosyltransferase resulted in significant increase in flavonols and anthocyanins content. The 4-fold increase in kaempferol content was primarily due to the glycosyltransferase specificity

## Methods

### Plant material and bacterial strains

Potato plants (*Solanum tuberosum *L. cv. Desiree) were obtained from "Saatzucht Fritz Lange KG" (Bad Schwartau, Germany). Control and transgenic plants were grown in the greenhouse in soil under 16 h light (22°C) and 8 h darkness (16°C) regime. Plants were grown in individual pots and were watered daily. Tubers were harvested after four months of growth and subsequently used for a field trial conducted near Wroclaw between May and September, 2005. All data presented in this paper pertain to tubers from field grown plants.

*Escherichia coli *strain DH5α (Bethesda Research Laboratories, Gaithersburg, USA) was cultivated using standard techniques [21]. *Agrobacterium tumefaciens *strain C58C1 containing plasmid pGV2260 [22] was cultivated in YEB medium [23].

### Generation of transgenic plants

In this study, two types of transgenic plants were used:

DFR 11, which over-expresses the *P. hybrida *cDNA encoding dihydroflavonol 4-reductase (EMBL/GenBank database acc no. X15537); and

DFR/UGT, which is a DFR 11 transgenic line [3] super-transformed with recently cloned glycosyltransferase cDNA from *S. sogarandinum *(EMBL/GenBank database acc no. AY033489).

Leaf explants were transformed using the pBin vector containing the appropriate cDNA in sense orientation. The cDNA was under the control of 35S promoter in DFR plants, or the tuber specific B33 promoter in DFR/UGT plants. In both lines, the cDNA was under the control of the OCS terminator. The *nptII *gene was used as a selection marker. The vector was introduced into the *A. tumefaciens *strain *C58C1: pGV2260 *[22]. Integration of the plasmid was verified by restriction enzyme analysis. Explants of young leaves of wild-type potato (*S. tuberosum *L. cv. Desiree) were transformed by immersing them in a suspension of *A. tumefaciens*. The inoculated leaf explants were then transferred to callus induction medium and shoot regeneration medium containing kanamycin (100 μg/ml). All selection media contained cefotaxime in order to eliminate any remaining *Agrobacterium *cells [22].

### Transgenic plant selection

The transformants were preselected by PCR and then selected by means of northern and western blot analysis.

Two-step PCR pre-selection was carried out with the following specific primers:

dihydroflavonol 4-reductase (DFR):

forward: GGTGCATTCTCTTTGCCACTTGC

reverse: GACAGTTTGCGTCACTGGAGCTG

glycosyltransferase (UGT):

forward: GTCCTCTTGGTGACATTTCCCACAC

reverse: TGAGGAAATGCCACCACAGGTACAC.

Genomic DNA isolated from three-week-old tissue-cultured plants was used as a template. Transgenic lines that tested positive for the predicted PCR products were subject to further analysis. The predicted products were 1143 bp long for the DFR gene, and 1100 bp long for the UGT gene. Final selection was carried using northern and western blot analysis. Total RNA was prepared from frozen young plant leaves or tubers using the guanidinium hydrochloride method [24]. The RNA was separated by electrophoresis on 1.5% (w/v) agarose containing 15% (v/v) formaldehyde. Then the RNA was transferred to a nylon membrane (Hybond N, Amersham, UK). The membrane was hybridized overnight at 42°C with radioactively labeled DFR cDNA and UGT cDNA as a probe. Then the filters were washed three times with SSPE buffer containing 0.1% SDS for 30 min at 42oC.

The fourteen transgenic lines that showed the highest levels of mRNA for UGT and DFR were finally selected by immunodetection. Potato tubers were homogenized in liquid N2. Total protein was extracted with buffer E containing 100 mM HEPES/NaOH (pH 7.4), 10 mM MgCl2, 2 mM EDTA (pH 8.0), 2 mM EGTA (pH 8.0), 1 mM PMSF, 0.2% Triton X-100, glycerol and 14 mM beta-mercaptoethanol. After centrifugation, proteins concentration was measured using the Bradford method (Bio-Rad Protein Microassay). The protein extracts prepared from the potato tubers were separated on a 12% SDS-polyacrylamide gel and electrophoretically transferred onto a nitrocellulose membrane (Schleicher and Schuell). Following transfer, the membrane was incubated with blocking buffer (5% dry milk), and then with antibody against 5-UGT-recombinant protein (1:2000 dilution) or with antibody against DFR-recombinant protein (1:3000 dilution). Goat anti-rabbit IgG conjugated with alkaline phosphatase was used as a secondary antibody at a dilution of 1:1500. The alkaline phosphatase was then stained with NBT (p-nitro blue tetrazolium chloride) and BCIP (5-bromo-4-chloro-3-indoyl-phosphate p-toluidide).

### DFR enzyme assay

Protein extracts were prepared from the potato tubers. Potato tubers were homogenized in liquid N_2_. Total protein was extracted with buffer E (100 mM HEPES/NaOH (pH 7.4), 10 mM MgCl_2_, 2 mM EDTA (pH 8.0), 2 mM EGTA (pH 8.0), 1 mM PMSF, 0,2% Triton X-100, glycerol, 14 mM beta-mercaptoethanol).

The extracts were centrifuged at 15,000 *g *for 15 min at 4°C, and the supernatant was used for further analysis. Protein concentration was measured using the Bradford method. DFR activity was measured in accordance with the procedure described by De-Yu [25]. The reaction mixture contained 25 mM Tris·HCl (pH 7.0), 4 mM NADPH, 100 μM taxifolin (dihydroquercetin), and protein extract. The reaction was initiated by addition of NADPH at 25°C, followed by measuring the rate of NADPH oxidation at 340 nm. The enzyme activity was calculated by using the absorption coefficient of NADPH (6.22 mM^-1^·cm^-1^). One unit of enzyme activity was equivalent to the oxidation of 1 μmol of NADPH per min.

### UGT enzyme assay

UGT activity was determined using the following standards as acceptor substrates: cinnamic acid, caffeic acid, coumaric acid, kaempferol, kaempferol-3-O-glycoside, kaempferol-5-O-glycoside, kaempferol-7-O-glycoside, 5-deoxykaempferol, peonidin chloride and peonidin-3-O-glycoside (TransMIT, Marburg, Germany). UDP-Glu was used as the donor of the sugar moiety. The reaction mixture (total volume, 75 μl) used for the assay of glycosyltransferase activity consisted of increasing content of aglycones (from 2 to 15 μg per sample, 1 mg/ml stock solution in ethylene glycol monomethyl ether) as the substrate, PBS buffer (pH 8.0), and 9 μl of UDP-Glu (10 nmol/μl). The reaction was carried out for 16 min at 30°C and was started by adding 50 μl (1 mg/ml) of enzyme solution. The phenolic acid and anthocyanin were then extracted with 500 μl of methanol, dried, re-dissolved in methanol (50 μl) and subjected to HPLC or UPLC analysis. Compounds were identified and quantified using standards.

Transgenic plants were selected using an assay mixture containing kaempferol as the substrate and tuber extract (30 μg) as the enzyme source.

### Extraction of phenolic compounds

150 mg of vacuum-dried tuber epidermis was extracted with 1 ml methanol-1% HCl solution in an ultrasonic bath for 15 min. After centrifugation supernatant was filtered through millipore (0.2 μm) then dried in a speedvac. Polyphenols were re-suspended in 1 ml methanol. The solution was then applied onto SPE column (Merck, Darmstadt, Germany). Retained compounds were eluted from the solid phase with 40% MeOH and analyzed on HPLC/UV.

### HPLC analysis of phenolics

Phenolics were analyzed using an HPLC system (Knauer, Germany) equipped with an automated sample injector and a UV detector (Knauer variable wavelength monitor type 87.00). The system was connected to a personal computer (HPLC Software/Hardware Package Version 2.21A). The sample extract was separated on a LiChroCART^® ^250-4 100 RP-18 (5 μm) column preceded by a LiChroCART^® ^4-4 100 RP-18 (5 μm) pre-column (Merck, Darmstadt, Germany). Compounds were detected by on-column measurement of UV absorption at 325 nm. The sensitivity was set at 0.04 a.u.f.s. The flow rate was adjusted to 1.0 ml/min. Phenolics were separated using a mobile phase consisting of the following components:

A: acetonitrile-formic acid (90:10 v/v); and

B: water-formic acid (90:10 v/v).

For the first two minutes, isocratic elution was carried out using 10% A in B. From 2 to 25 minutes, a linear gradient was applied using 10 to 30% A in B. From 25 to 27 minutes, a linear gradient was applied using 30 to 70% A in B. The volume of the sample injected was 20 μl. Calibration graphs of polyphenols were prepared by measuring the areas under the peaks. The graphs were linear in the range examined (0.02 to 0.10 mg/ml). For the HPLC analyses, gradient grade acetonitrile was used. Water was glass distilled and deionized. Solvent solutions were vacuum degassed with sonication before use. All experiments were performed at room temperature (20°C). Compounds were identified and quantified using standards.

### Analysis of the substrate specificity of recombinant UGT by UPLC

The products of the reaction of recombinant UGT with different substrates were analyzed using the Acquity UPLC system (Waters). A 2 μl sample was applied to an Acquity UPLC BEH C18 column (2.1 × 100 mm, 1.7 μm). The mobile phase was passed through the column at a flow rate of 0.4 ml/min. The mobile phase consisted of the following components:

A: 50 mM aqueous ammonium formate (pH 3.0); and

B: 100% acetonitrile.

The column was eluted for 1 min with 80% A in B, for 4 min with 60% A in B, and for 6 min with 80% A in B. The column was kept at room temperature. A photodiode array (PDA) was used to detect UV visible absorption between 210 and 500 nm. Compounds were identified and quantified using standards.

### Determination of antioxidant capacity

Antioxidant activity in the extracts was measured using chemiluminescence. 10 mg of potato tuber epidermis was extracted with methanol. The extract was diluted from 1,000 to 15,000 times with water and directly analyzed. The experiments were performed in a final volume of 250 μl on white microplates in a freshly prepared solution containing 0.1 M Tris-HCl buffer (pH 9.0) and 4 mM AAPH (2,2'-azobis(2-amidinopropane) dihydrochloride). The luminol solution (100 μM) and diluted extracts were automatically injected. The photons produced in the reaction were counted on an EG&G Berthold LB96P microplate luminometer at 30°C. The antioxidant potential was defined as the amount of tuber extract that inhibits luminol chemiluminescence by 50% and was expressed as IC_50_.

### Determination of proanthocyanins

The method used was based on the fact that condensed tannins give rise to anthocyanins when heated in mineral acid. Dried tuber peels (100 mg) were extracted with 7 ml of n-BuOH-conc. HCl (95:5, v/v) and 0.2 ml of a 2% (w/v) NH4Fe(SO4)2 × 12 H20 in 2 M HCl. The mixture was incubated at 95°C for 40 min. After centrifugation, the absorbance at 550 nm measured. Proanthocyanin content was calculated using catechin as standard (A^1% ^at 550 nm = 280).

### TBARS determination

The level of thiobarbituric acid-reactive substances (TBARS) in the samples was determined [26]. Oil samples (1 μl) were oxidized at 140°C for up to 40 min with or without (2 μl) potato extract (10 mg vacuum-dried tuber epidermis was subjected to acidic-methanol extraction). Two ml of reagent A (15% trichloroacetic acid and 0.37% thiobarbituric acid in 0.25 M HCl) was added and the mixture was thoroughly blended. Test tubes containing the samples were stopped with glass marbles, heated at 100°C for 15 min, cooled under running tap water, and centrifuged for 10 min at 2,000 g. The absorbance was measured at 535 nm using a spectrophotometer (Cecil CE-2020). The reference blank contained the TBA reagent.

The oil from flaxseeds used for reaction was prepared in accordance with the method described by Allen and Good [27]. Briefly, 1 g of seeds was ground in a mortar with 1 ml of water. The homogenate was suspended in 2 ml of methanol and 4 ml of chloroform. 3.5 ml of 0.9% NaCl was then added. The mixture was gently agitated and left to settle for 24 h at room temperature. The lower chloroform phase was collected and the extraction was repeated. The chloroform was evaporated using a rotary vacuum evaporator. The lipids were resuspended in chloroform/methanol (1:2 v/v) and stored at -20°C.

### Analysis of lignin content

10 mg of vacuum-dried tuber epidermis was suspended in 10 ml of water and heated for 60 min at 65°C. The sample was then filtered through a GF/A glass fiber filter and sequentially rinsed in water, ethanol, acetone and diethyl ether. The filter was then placed in a glass vial and heated overnight at 70°C. 2.5 ml of 25% acetyl bromide was added, and the vial was heated for 2 h at 50°C. After cooling of the samples 10 ml of 2 N sodium hydroxide and 1 ml of acetic acid was added. After incubation overnight at room temperature, the absorbance at 280 nm was measured. A calibration curve was constructed using coniferyl alcohol as the standard. Results were expressed in terms of equivalents of coniferyl alcohol.

### Statistical analysis

Data on the parameters measured were statistically elaborated using ANOVA and the Laven test, followed by the RIR Tukey or Kruskal-Wallis test. All calculations were carried out using the STATISTICA 7.1 software package (StatSoft Polska, Poland).

## Authors' contributions

AAS constructed and selected the transgenic plants, and performed the experiments and the statistical analysis. AKS participated in the HPLC analysis. AK carried out the UPLC analysis and helped draft the manuscript. JS conceived of the study, and participated in its design and coordination. All authors read and approved the final manuscript.
